# Supplementary material for: Widefield Diamond Quantum Sensing with Neuromorphic Vision Sensors
Source: Adv Sci (Weinh). 2023 Nov 8;11(2):2304355. doi: 10.1002/advs.202304355 (PMC10787069; doi:10.1002/advs.202304355)
Supplement: Supplementary file 1 — Supporting Information [file ADVS-11-2304355-s001.pdf]

ADVANCED SCIENCE
Open Access

Supporting Information

for *Adv. Sci.*, DOI 10.1002/advs.202304355

Widefield Diamond Quantum Sensing with Neuromorphic Vision Sensors

Zhiyuan Du, Madhav Gupta, Feng Xu, Kai Zhang, Jiahua Zhang, Yan Zhou, Yiyao Liu, Zhenyu Wang, Jörg Wrachtrup, Ngai Wong, Can Li* and Zhiqin Chu**

Supporting Information

Widefield diamond quantum sensing with neuromorphic vision sensors

Zhiyuan Du†, Madhav Gupta†, Feng Xu†, Kai Zhang, Jiahua Zhang, Yan Zhou, Yiyao Liu, Zhenyu Wang, Jörg Wrachtrup, Ngai Wong, Can Li,* and Zhiqin Chu**

Z. Du, M. Gupta, F. Xu, K. Zhang, J. Zhang, N. Wong, C. Li, Z. Chu
Department of Electrical and Electronic Engineering
The University of Hong Kong
Hong Kong 999077, P. R. China
E-mail: nwong@eee.hku.hk; canl@hku.hk; zqchu@eee.hku.hk;

K. Zhang, Y. Zhou
School of Science and Engineering
The Chinese University of Hong Kong, Shenzhen
Shenzhen 518000, China

Y. Liu, Z. Wang
Guangdong Provincial Key Laboratory of Quantum Engineering and Quantum Materials
School of Physics and Telecommunication Engineering
South China Normal University
Guangzhou 510006, China

Z. Wang
Frontier Research Institute for Physics
South China Normal University
Guangzhou 510006, China

Jörg Wrachtrup
3rd Institute of Physics, Research Center SCoPE and IQST
University of Stuttgart
Stuttgart 70569, Germany

Jörg Wrachtrup
Max Planck Institute for Solid State Research
Stuttgart 70569, Germany

Z. Chu
School of Biomedical Sciences
The University of Hong Kong
Hong Kong 999077, P. R. China

Z. Chu
Advanced Biomedical Instrumentation Centre
Hong Kong Science Park
Hong Kong 999077, P. R. China
[†]These authors have contributed equally to this work.

Section 1. Supporting Figures for Experimental Section

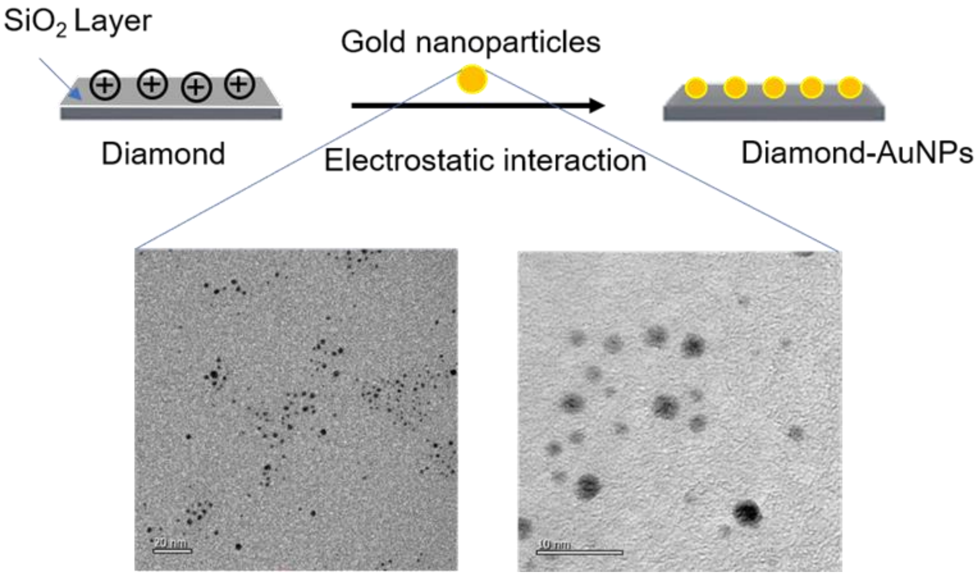

Figure S1. Fabrication process of gold particles on diamond. Negatively charged gold nanoparticles can be adsorbed on the surface of the diamond with modified silicon layer by electrostatic interaction.

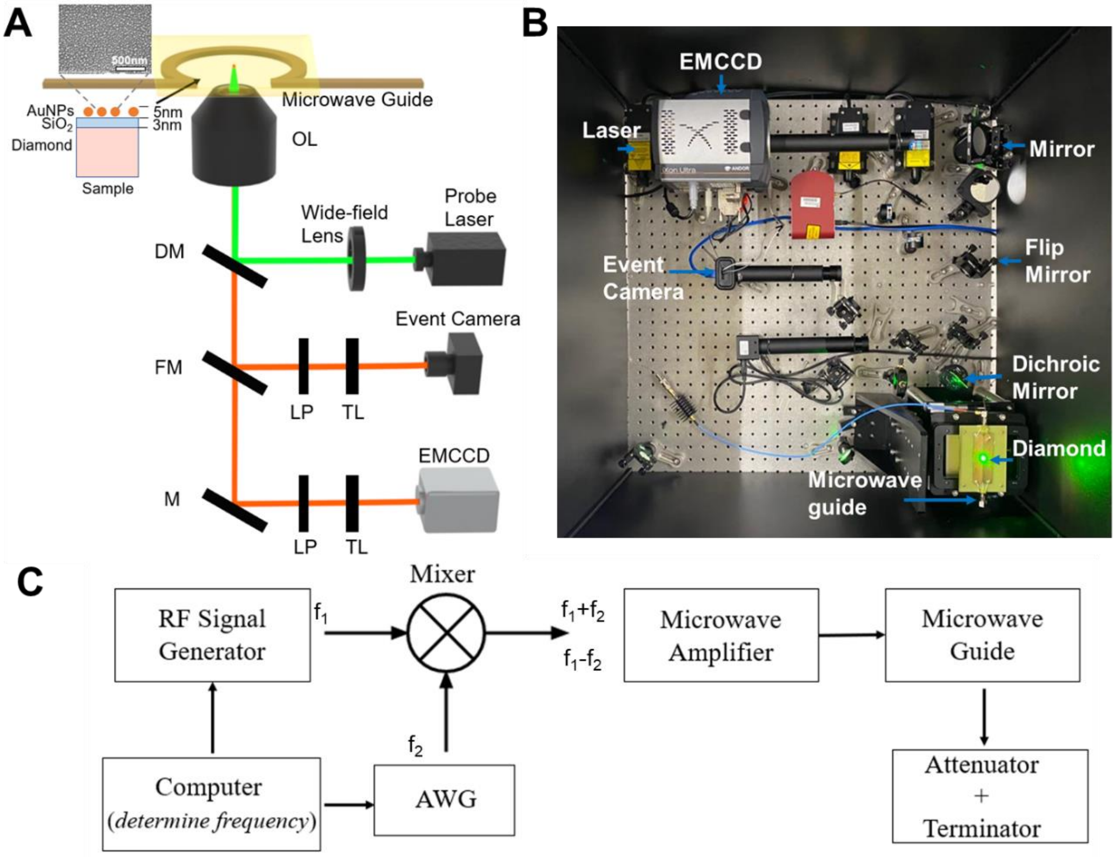

Figure S2. Measurement Setup. A) Experimental set up: a 532nm green laser source delivers probe light onto the diamond sample. The diamond sample contains a 3 nm SiO_2 layer and randomly-distributed gold nanoparticles (AuNPs) for the temperature measurement experiment shown in Section 5. Tuned by the fed microwave through a MW guide, the emitted fluorescence is measured by an event camera or an EMCCD (switched by a flip mirror, FM) after passing the optical system (including DM: dichroic mirror, cut-off wavelength 605nm; LP: long-pass filter, cut-off wavelength 650nm; TL: tube lens). B) Picture of the experiment set-up taken in the lab; C). System schematic for generating frequency-swept micro-wave signal. AWG: arbitrary waveform generator.

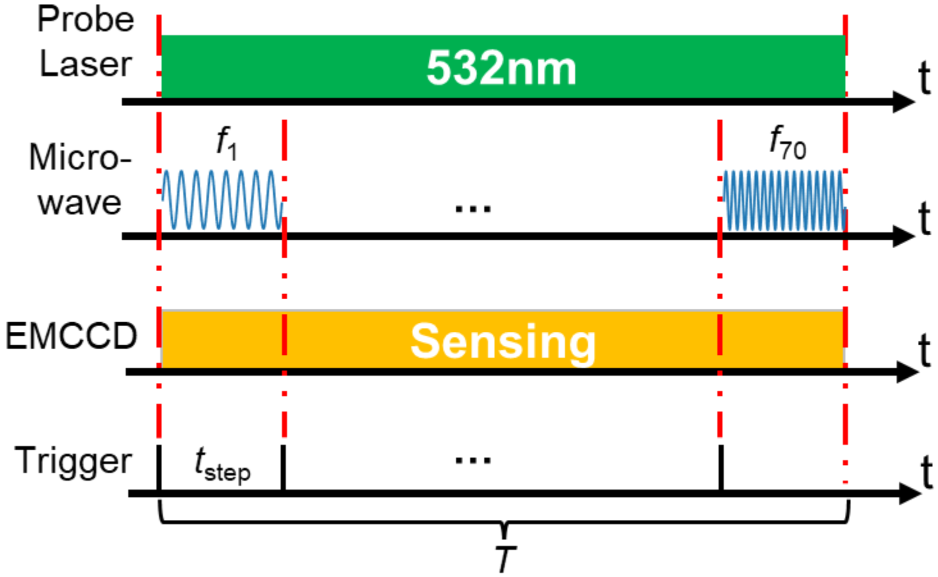

Figure S3. Experiment procedure for frame-based ODMR. The 532nm laser is kept on to excite the spins for performing continuous-wave ODMR. Microwave (MW) is swept from f_1-2836MHz to f_{70}-2905MHz discretely with a step size of 1MHz (time duration is t_{step}). The fluorescence intensity is recorded by the EMCCD, synchronized with the MW frequency sweep through external trigger pulses.

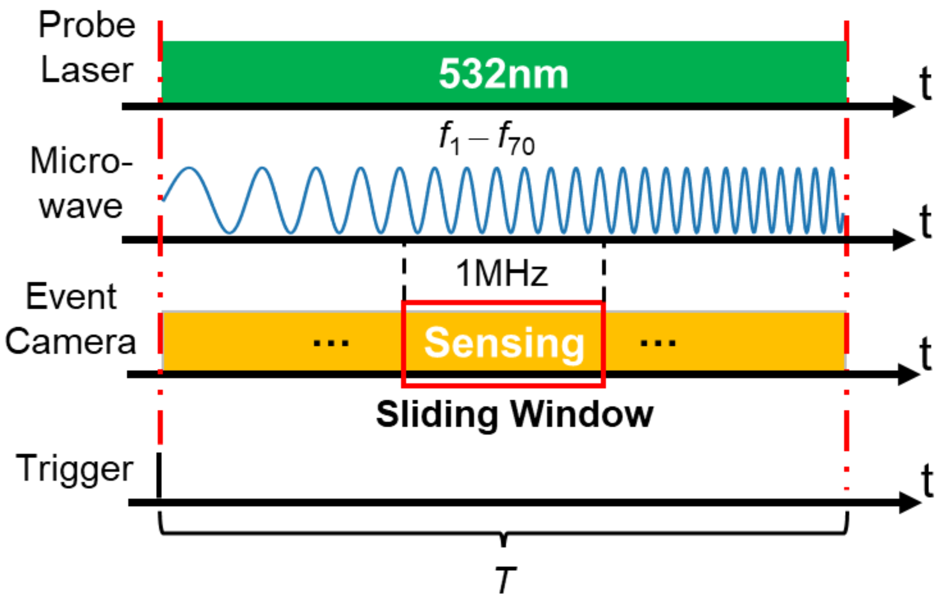

Figure S4. Experiment procedure for event-based ODMR. The 532nm laser is kept on to excite the spins for performing continuous-wave ODMR. For the event-based ODMR, the MW frequency changes linearly from f_1-2836MHz to f_{70}-2905MHz with time T, during which the fluorescence change is continuously detected by the event camera. A frequency window covering 1MHz slides across the output events during which, the event value within the window is summed to represent the process results of its central frequency. To reduce the influence of noise events, the measurement process is repeated for 10 loops and the results are accumulated together.

Section 2. Deviation and Correction of Reconstructed f_0.

We attribute the deviation shown in Figure 3 to two reasons. The first is the delay between AWG and event camera. As shown in Figure S5A, suppose the event camera starts detecting events at t_{e0}, t_{e1}, t_{e2}…, while the AWG starts tuning frequency at t_{a0}, t_{a1}, t_{a2}…during the repeated sweeps. Then it is obvious that there will be a frequency difference (Δf=f_{e0}- f_{a0}) after mapping the event time back to frequency. Suppose the sweeping speed is Δt/MHz, the time delay t_{ai}- t_{ei} will cause a frequency deviation as $\Delta f = (t_{ai} - t_{ei})$ /Δt MHz.

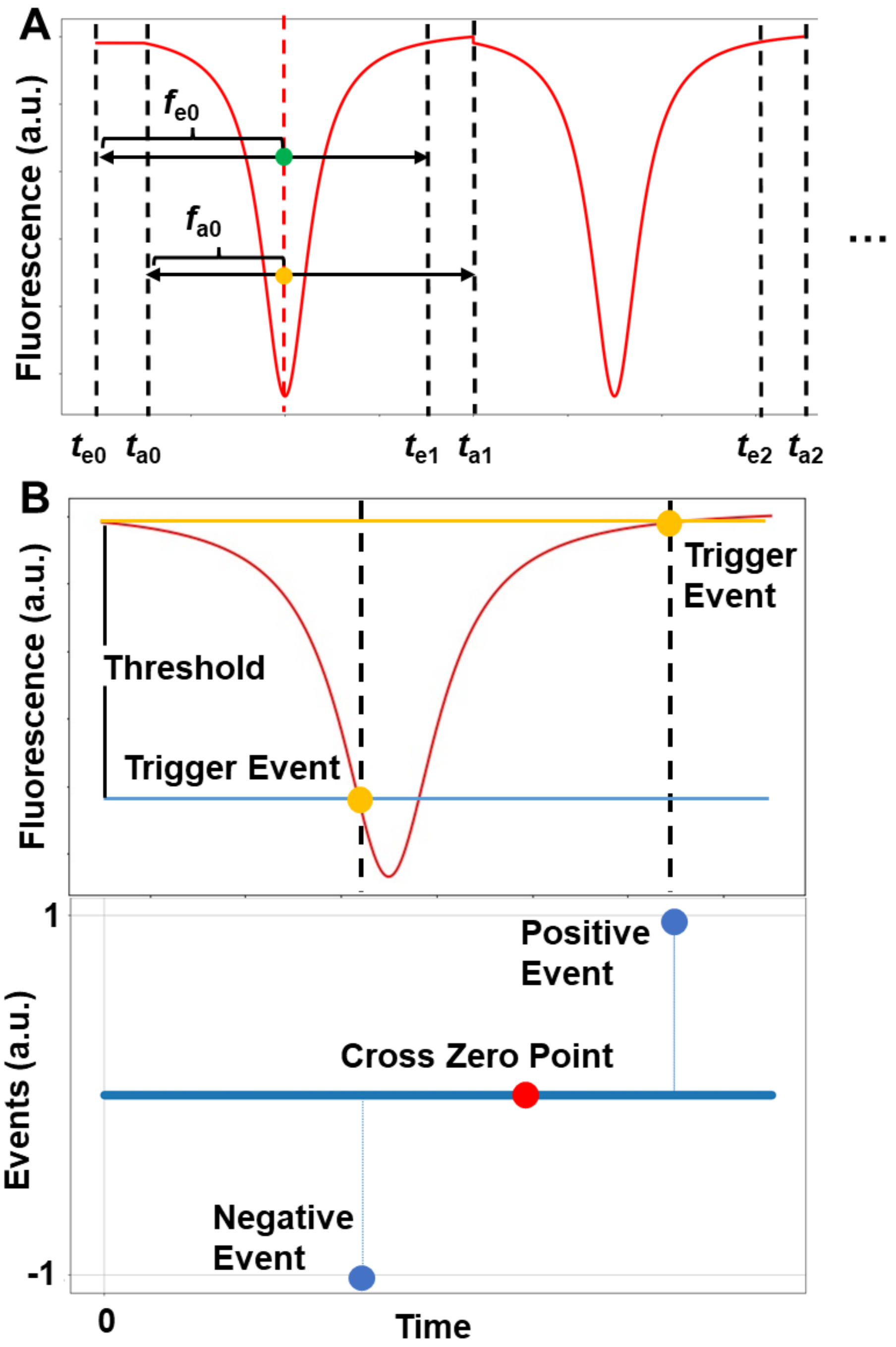

Figure S5. Scheme showing deviation caused by time delay and the large threshold of the event camera. A) Lorentzian function vs time representing the fluorescence spectrum of NV center with frequency sweeping. t_{e0}, t_{e1}, t_{e2} represent the time when event camera starts sampling, while t_{a0}, t_{a1}, t_{a2} is the time when AWG starts sweeping frequency. B) Upper panel: Lorentzian function vs time representing the fluorescence spectrum of NV center with frequency sweeping. Lower panel: events produced when the fluorescence change reaches the threshold. The cross zero point is calculated by the moving sum method with appropriate window covering the two events, which is also the resonance position of reconstructed derivative spectrum.

Besides the time delay, it can be seen in Figure S5B that the large threshold (chosen to suppress background noise) will also bring deviation. With a little exaggeration for clear explanation, we show a large threshold on the fluorescence spectrum (upper panel), then two events with conversed polarity will be produced as the frequency is swept. It is obvious that after moving sum process, the cross-zero position which is also the resonance position of our method will be located between the two events. Comparing with the real resonance position (the dip in the upper panel), one can find an obvious deviation.

It is worth noting that the deviation caused by both two reasons are symmetric, which means if the frequency is swept in a descending direction, the deviated resonance position will shift to the left side of the real resonance position. From table S1, it can be demonstrated the deviation could be corrected by taking forward and backward sweeping and taking average of the reconstructed resonance frequency.

Table S1. Resonance frequency f_0 extracted by different methods, including forward sweeping, backward sweeping and their average, compared with the result of EMCCD. The deviation of single direction sweeping is canceled by averaging the results by forward and backward sweeping.

Method	Forward Sweeping	Backward Sweeping	Average	EMCCD
f_0 (MHz)	2872.23 (±0.03)	2867.03 (±0.03)	2869.63 (±0.03)	2869.62 (±0.03)

Section3. ODMR spectrums from repeated measurements

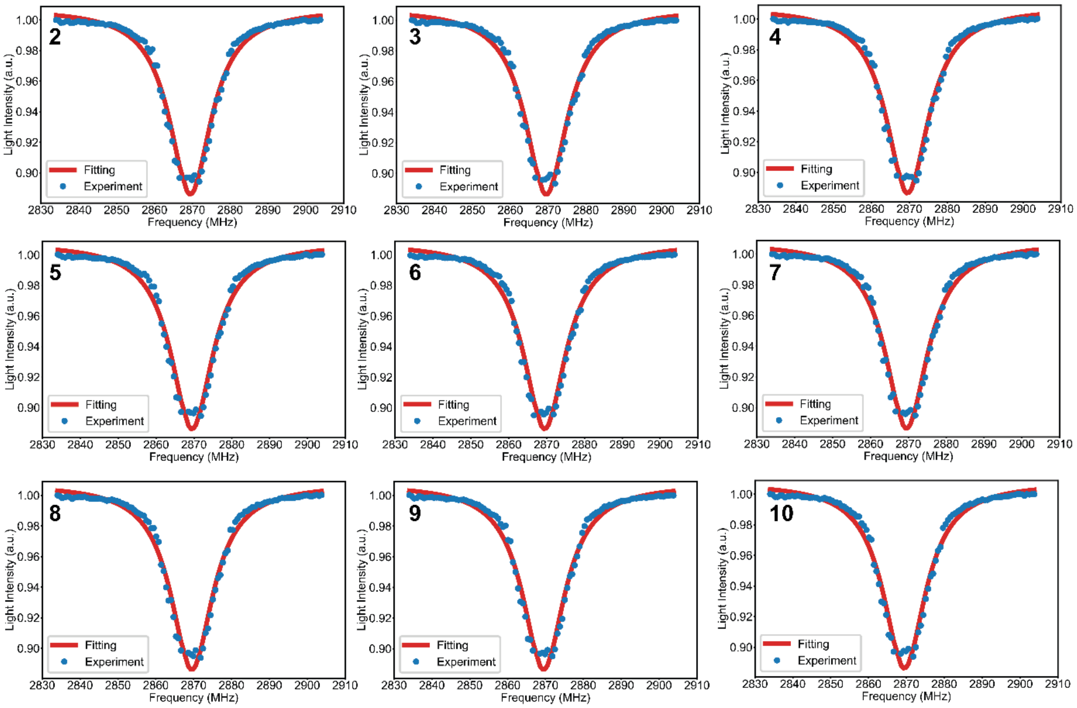

Figure S6. The other 9 Lorentzian spectrums reconstructed from repeated frame-based ODMR

measurement with the same experimental conditions as Figure. 3 E in main text. The standard deviation is calculated based on the fitted resonance frequencies.

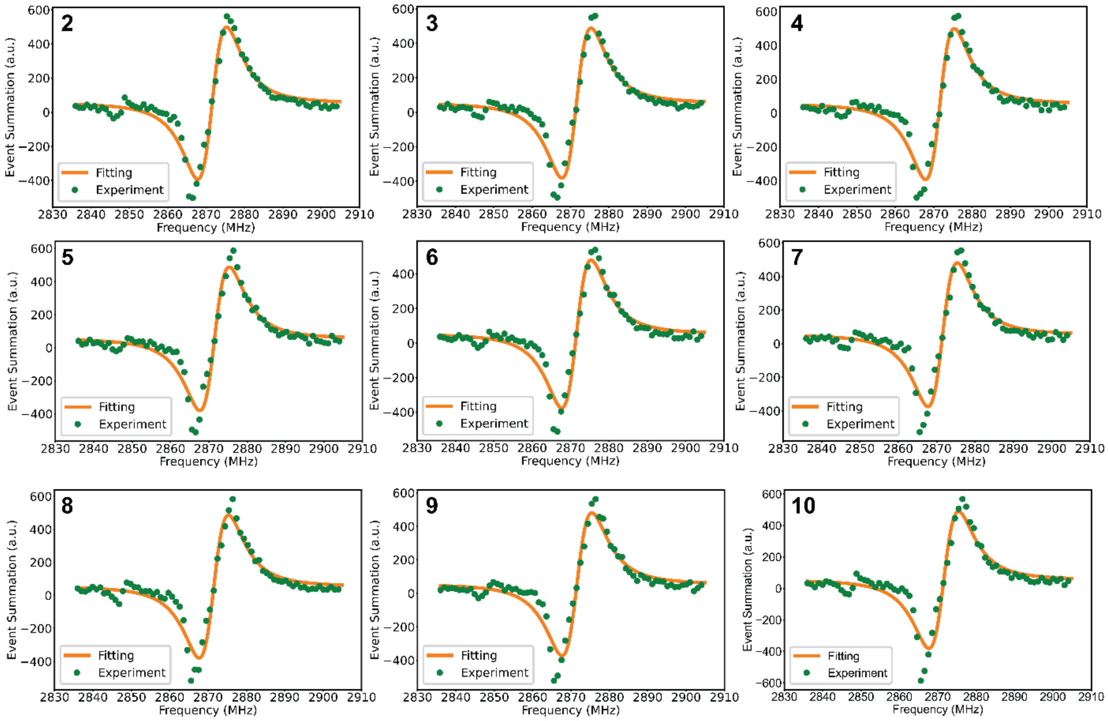

Figure S7. The other 9 derivative Lorentzian spectrums reconstructed from repeated event-based ODMR measurement with the same experimental conditions as Figure. 3 F in main text. The standard deviation is calculated based on the fitted resonance frequencies.

Section 4. Sampling Model

For frame-based ODMR, the precision is inversely related with the square root of exposure time. Event camera, however, works differently to frame-based cameras, which only measures the change of photo-current rather than integrating photo-generated charges, and as a result is not limited by the exposure time (or the concept 'exposure time' does not apply to an event camera). Therefore, there must be a different mechanism that affects the change of precision.

As the resonance frequency f_0 is extracted from the summed events, the raw event data should determine the variation of f_0. To figure out the underlying physics, we measured the number of recorded events within the laser spot (20 by 20 pixels) during repeated sweeping (cf. Figure S8A) and also checked the temporal event response under different sweeping periods (Figure S8B). The event numbers shown in Figure S8A are normalized as $N_{avg} = \frac{N_{events}}{N_{pixels} * N_{loops}}$, where N_{events}, N_{avg} are the total and averaged event number while N_{pixels}, N_{loops} are the number of pixels and sweep loops of which the events are counted. It is first found that quite a few events are produced during every single sweeping. As is shown in Figure S8A, the averaged event number is even smaller than 2 (as analyzed in the following part, ideally there should be at least one negative and one positive event during each sweep) when sensing time is shorter than 0.3s,

which means for some sweeps, null events are produced. This can be verified from the temporal event spike plots as showed in No. ⑥ of Figure S8B. Indeed, even for the long sensing time (lager than 0.3s), like No. ①-③ in Figure S8B, some events are irregular which could be regarded as noise events. In other words, the events are quite sparse in time domain and the faster the sweep, the sparser the events. As we have mentioned in the main text, the event density describes the gradient of Lorentzian spectrum. When the events are too sparse, the summed event value will deviate from the expected level, which makes the Lorentzian fitting imprecise.

We build a sampling model for explaining the decrease of event number (see Figure S9), where the fluorescence spectrum is considered as a Lorentzian curve (according to the photo-physics of NV centers) mixed with photon shot noise. According to the working process of an event camera, events will be triggered when the intensity change is larger than the threshold. Here, we set a large threshold so that events are only produced near the dip and plateau during each sweep. This equals to sampling at the dip and plateau while the superposition of multiple sweeping represents the statistical distribution of the dip and plateau of the spectrum. Due to the large threshold value (higher than the contrast of the Lorentzian spectrum), null events are produced during some sweepings (see both No.⑥ of Figure S8B and Figure S9). Those null-events sweeps do not contribute to the final deduce of f_0. And the faster the sweep (the shorter the sweeping period), more null sweeps appear, for which the variation/shift of reconstructed spectrum is more obvious, i.e. the larger the precision of fitted f_0. Based on this model, we carried out Monte Carlo simulations with different periods. The calculated precision (Figure S10) shows a similar relationship with period compared with those in experiments ($\sigma_{simulation} = 0.028\frac{1}{\tau^{0.45}}$ vs $\sigma_{experiment} = 0.023\frac{1}{\tau^{0.43}}$).

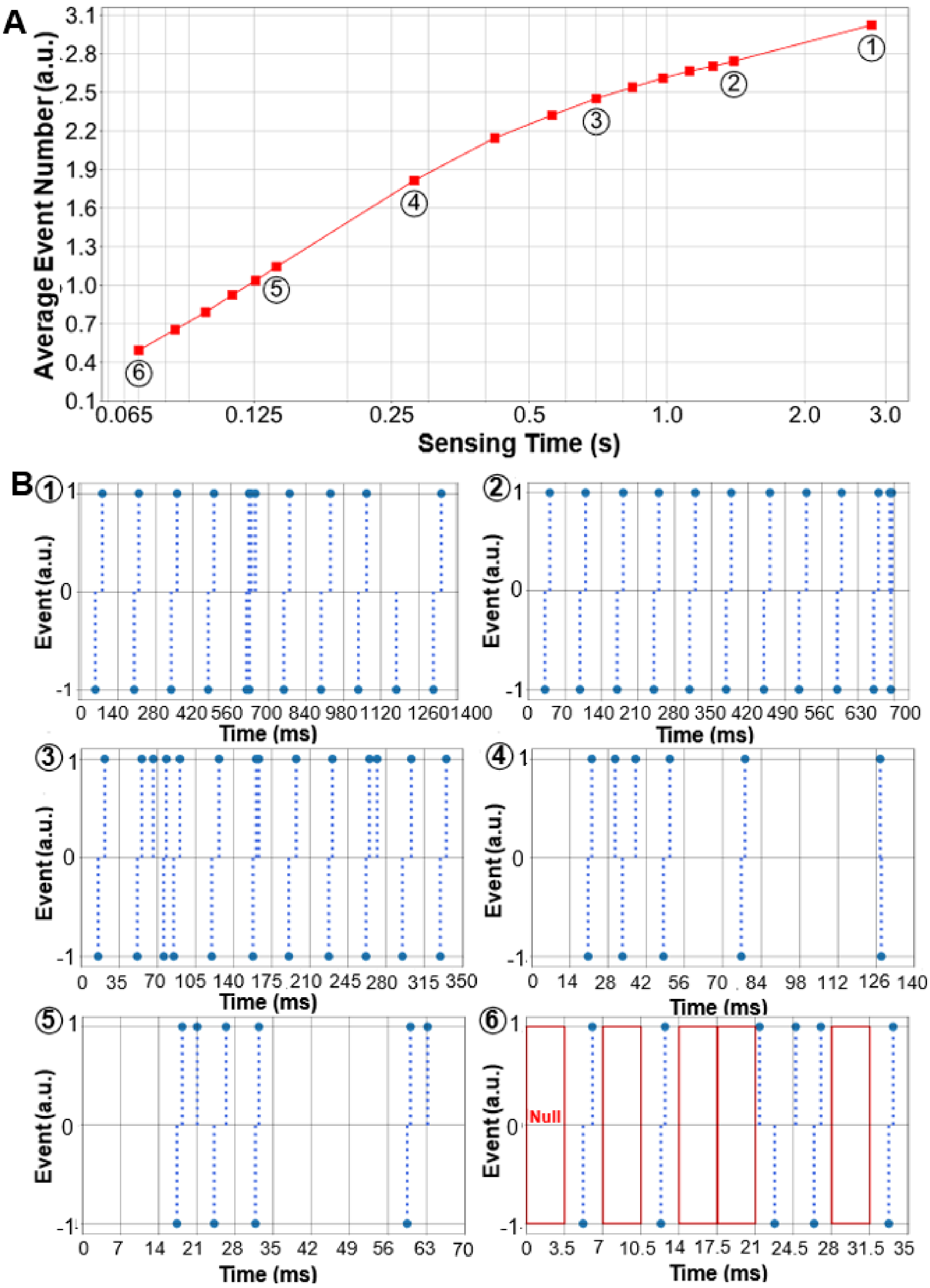

Figure S8. Event number counting. A) average event number (per pixel per sweeping loop) counted for different sensing time (i.e. different sweeping speed of MW frequency); The sensing time considers both forward and backward sweep. B) Temporal event spikes measured for different sensing times chosen in A) (taken from only forward sweep for clear and simple illustration). The spikes are valued +1 (positive event) and -1 (negative event). The x-axis grids

show cycled sweep loops. For sub-figure 6, those null sweep loops with no events produced are labelled with red boxes.

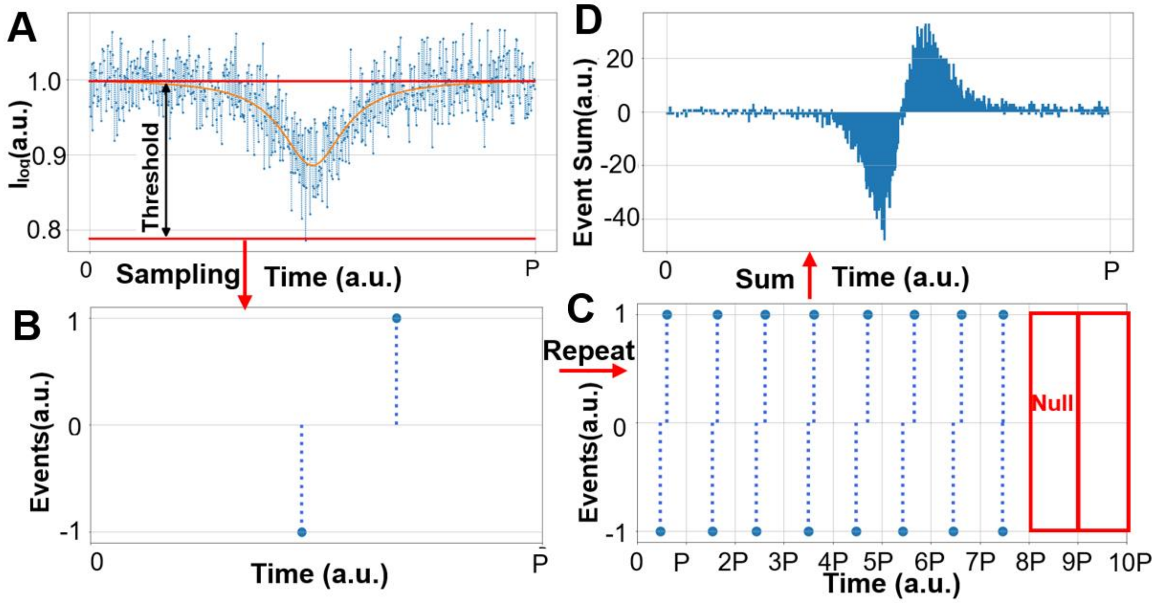

Figure S9. Sampling model. A) Lorentzian function mixed with Poisson noise representing the photo-current spectrum during ODMR measurement. According to the event camera's working principle, the photo-current is transformed in a logarithm style, and a large threshold is shown for sampling the spectrum change; B) Events generated by sampling spectrum with a large threshold in A); C) Events produced by repeated sampling (as done in our experiment). The null event sweeps are also labelled with red boxes; D) Accumulated events of total 4000 sweep loops.

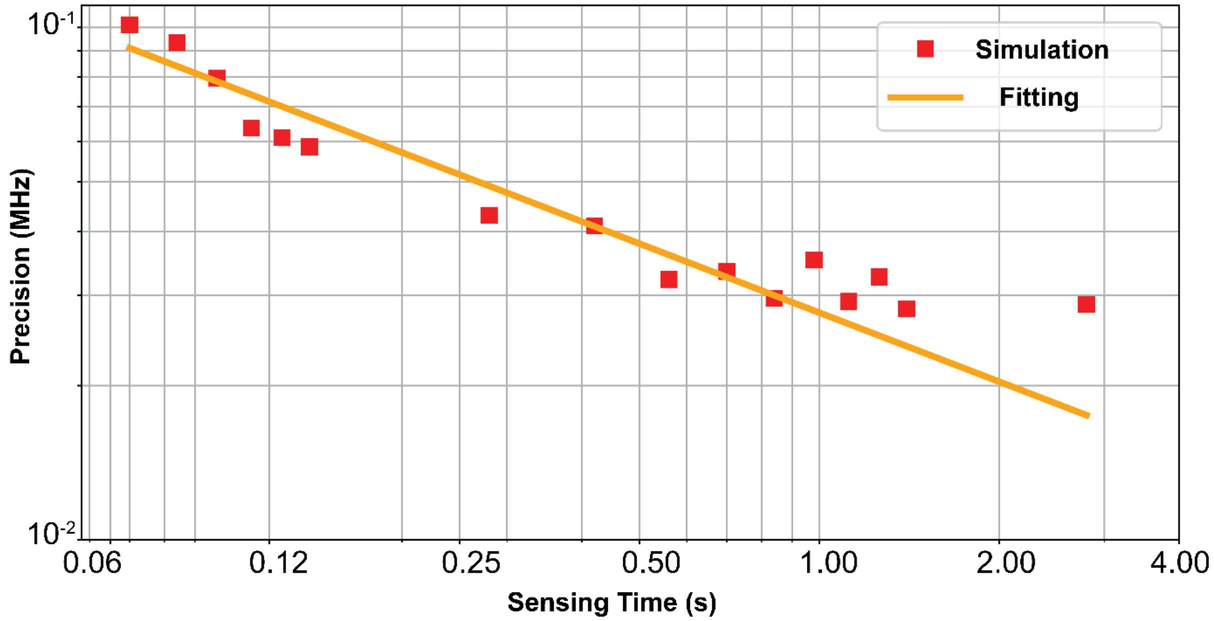

Figure S10. Simulation results**.** Precision σ changes with increasing sensing time τ produced by Monte Carlo simulation. The fitted relationship is expressed as: $\sigma_{simulation} = 0.028\frac{1}{\tau^{0.45}}$.

Section 5. Adaptivity Analysis

A superiority of our method is the adaptive sensing process, in both time and space domains. To quantitatively describe this adaptivity, we calculated the spatial and temporal signal-to-background ratios (SBR_t and SBR_s)[1], respectively. During the frequency sweep, the fluorescence change is sampled with adaptive time interval by the event camera. We therefore calculated the event density (Figure S11), defined as the temporally averaged event number along with the frequency sweep (the event numbers of 20*20 pixels are accounted to relieve the randomness). As expected, the event density is low at the beginning and ending of frequency sweep corresponding to the smooth parts of fluorescence spectrum, while it shows two peaks around the resonance frequency. The temporal SBR_t is then defined as ratio of top event density Np over the background value (here we use the smallest event density). The SBR_t is 10 for our event-based method (corresponding to measurement in Figure 3) in comparison with 1 for the frame-based measurement (the EMCCD output speed is unchanged as the fluorescence intensity is recorded with constant time interval).

Similar SBR analysis can also be performed for the space domain, where the signal and background are represented by the light intensity (for EMCCD) /event number (for event camera) from the ROI (i.e. NV located) and background pixels respectively. Specifically, we draw a line crossing the center of laser spot and sum the light intensity/ event number of 20*20 pixels alongside, as shown in Figure S12A and Figure S12B, respectively. Again, the ratio between the top and bottom value are computed as the SBR_s, where a result of 194 vs 64 is obtained for the event-based and frame-based method. An obviously higher SBRs is seen in our method due to the special event generate process being suppressed in the background pixels where there is no significant fluorescence change. In contrast, traditional EMCCD cannot adaptively distinguish the ROI and background signal for which both useful signal and background noise are recorded. The calculated results (SBR_t and SBR_s) are listed in Table 1 in the main text for quantitative comparison.

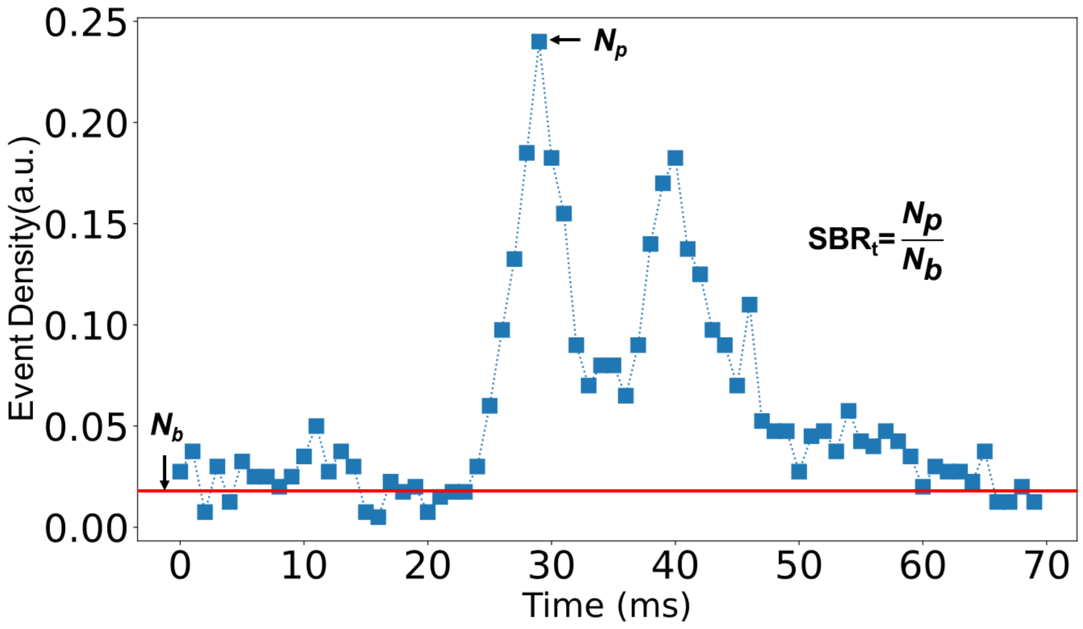

Figure S11. Event density changed during MW frequency sweeping. The events are counted from the central 20*20 pixels in the laser spot during every 1ms temporal range and then averaged by the pixel number (400). Based on the result, the temporal signal-to-background ratio (SBR_t) is defined and calculated.

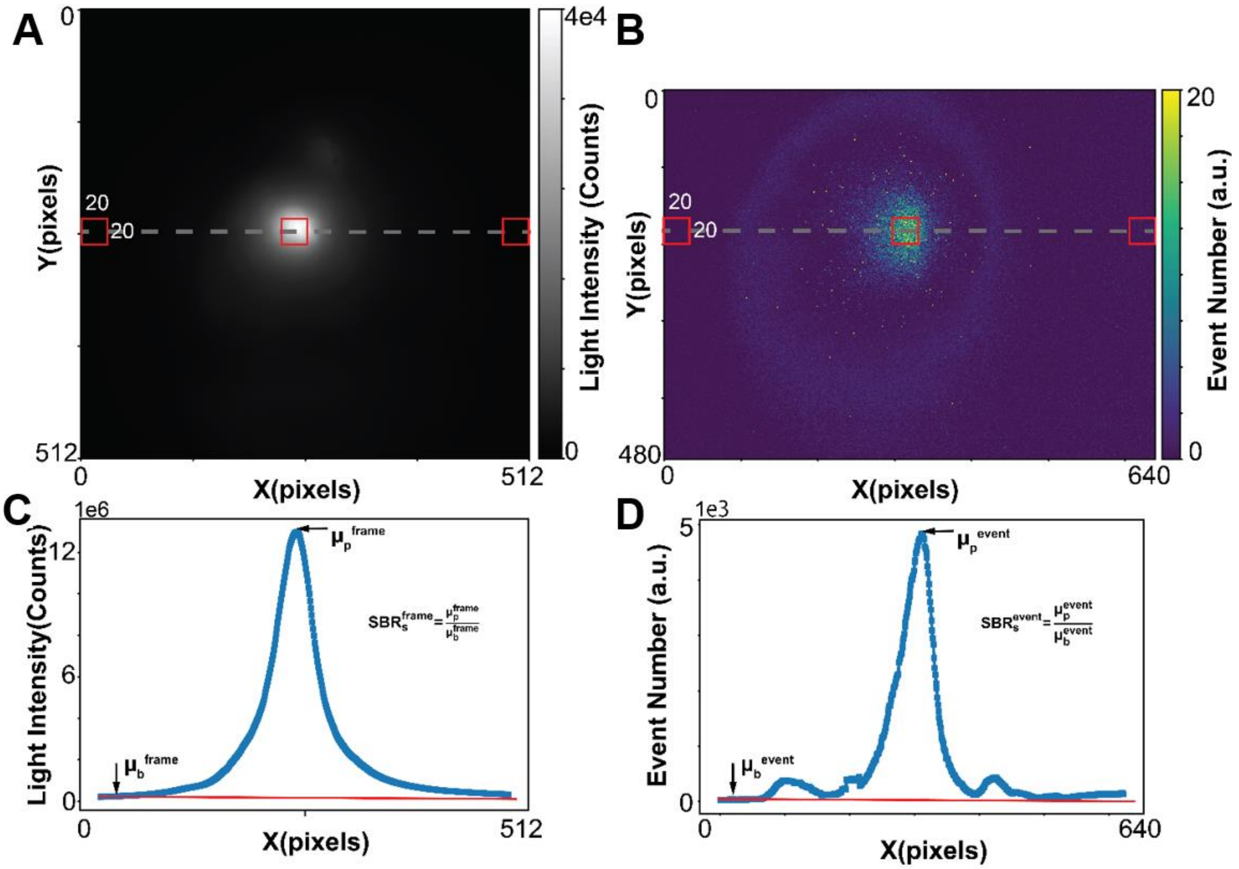

Figure S12. Comparison of SBR_s for frame-based ODMR and event-based ODMR. A) The frame recording the fluorescence intensity measured with EMCCD at *f*=2836MHz. B) Recorded event number across the frame of event camera during MW frequency sweep for 10 loops (sweeping period is 7ms). C), D) Light intensity and Event number counted along the dashed line in (A) and (B). The value is the summation of signals belonging to the nearby 20*20 pixels. Based on the results, the spatial signal-to-background ratio (SBR_s) is defined and calculated.

Section 6. Dynamic Temperature Measurement

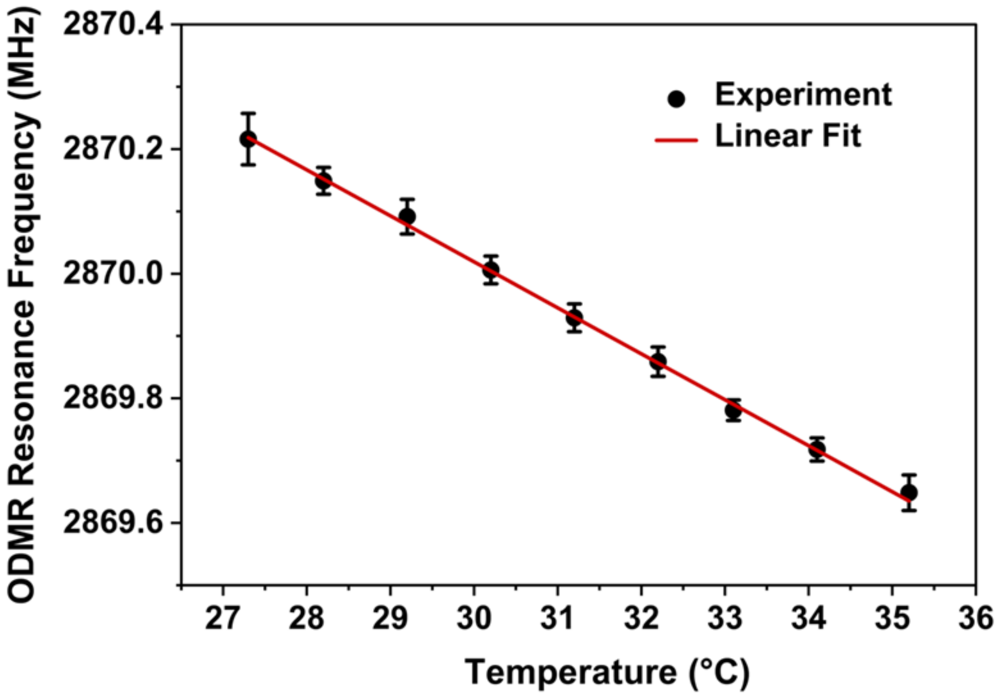

Figure. S13. ODMR Resonance Frequency measured using EMCCD as a function of temperature. A thermal susceptibility of 74 kHz/°C is extracted.

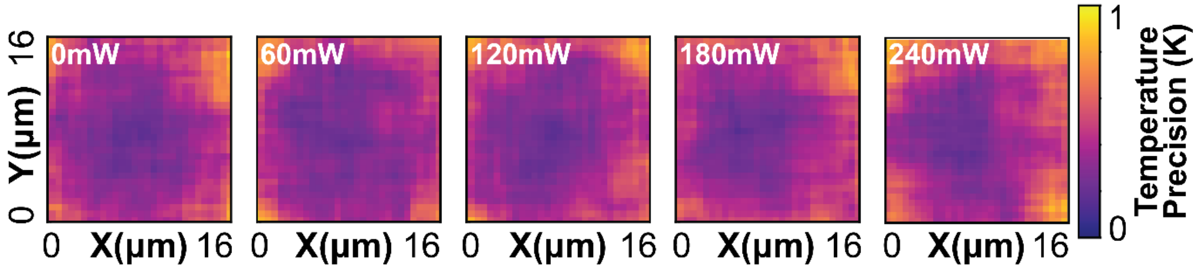

Figure. S14. Temperature precision distribution corresponding to static temperature measurement in Figure 4B. The standard deviation of temperatures from 10 repeated measurements is calculated to represent sensing precision.

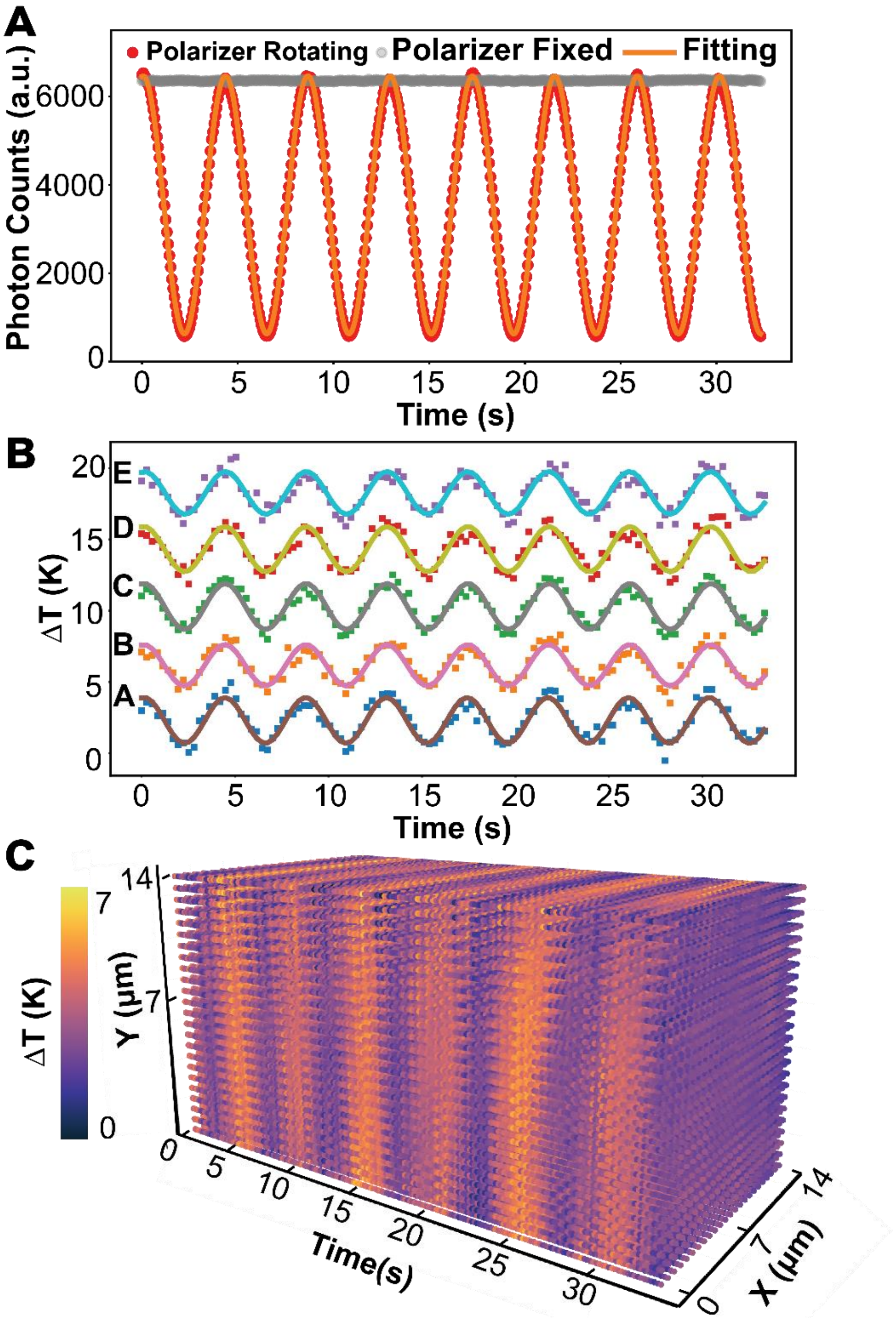

Figure S15. A) Measured laser power tuned by the linear polarizer (Figure 4A) with the axis rotated continuously (red dots) and fixed at the polarization direction of laser (gray dots). The tuned laser power vs time is fitted with a cosine square function. B) Temporal temperature response taken out from different pixels in Figure 4C (A (15,5), B (15,15), C (15,25), D (5,15), E (25,15) @ 60 by 60 pixels frame). For a clear display, the temperatures are biased by N*4 K, where N=0-5 indicates points A-E. C) Spatiotemporal temperature response measured by the event-based ODMR with a sweep period of 7ms (i.e. the temporal resolution of temperature sensing is 0.14s). For a clear display, we show here a smaller ROI (14 by 14 μm^2).

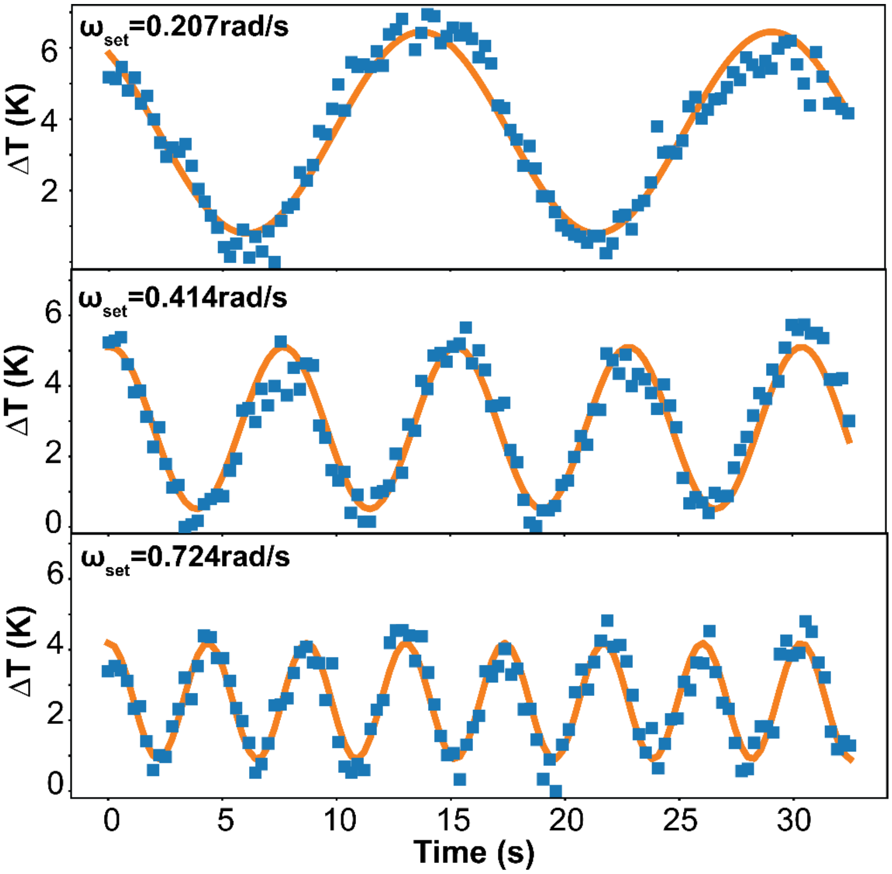

Figure S16. Dynamic temperature measurement under different modulation speed with the event-based method. The temperature change is measured with a 0.28s temporal resolution when the polarizer rotates at different speeds from ω_{set}=0.207 rad/s to ω_{set}=0.724 rad/s.

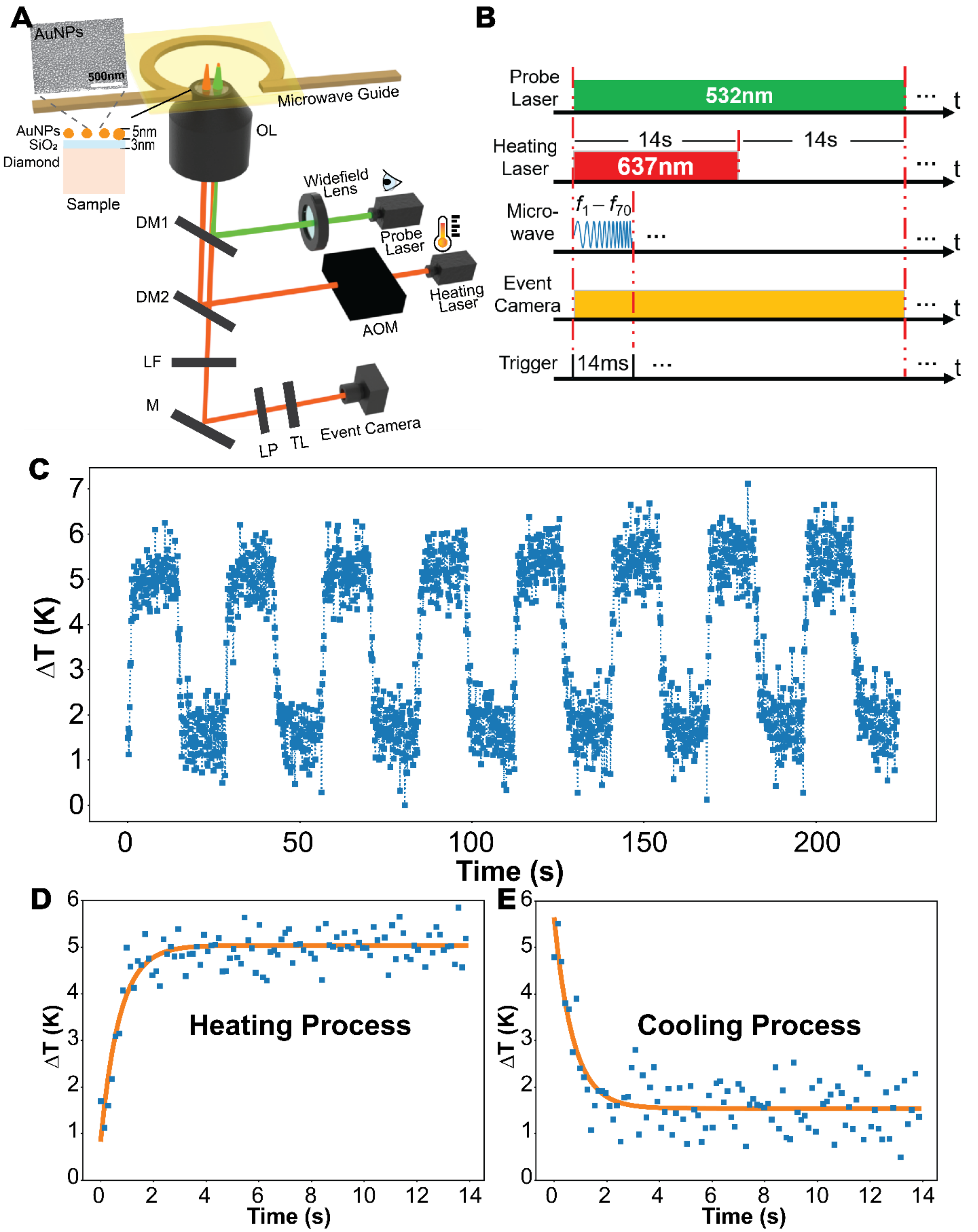

Figure S17. Transition temperature measurement. A) Set-up for transition temperature measurement where an AOM is added to the old system to switch the red laser (the switch time is around 10ns). B). Protocol for performing the temperature measurement. The green laser is kept

on for exciting the spin, during which the red laser is switched on and off every 28s. Trigger signals are sent to AWG and event camera every 14ms to trigger periodic ODMR measurements. C) Periodic temperature switch measured based on the protocol in (B). D) and E) Zoomed results taken from the first cycle in (C), illustrating the heating process and the cooling process, respectively. The fitted functions for (D) and (E) are $-4.16 * \exp\left(-\frac{t}{0.71}\right) + 5.04$ and $4.05 * \exp\left(-\frac{t}{0.71}\right) + 1.54$, respectively.

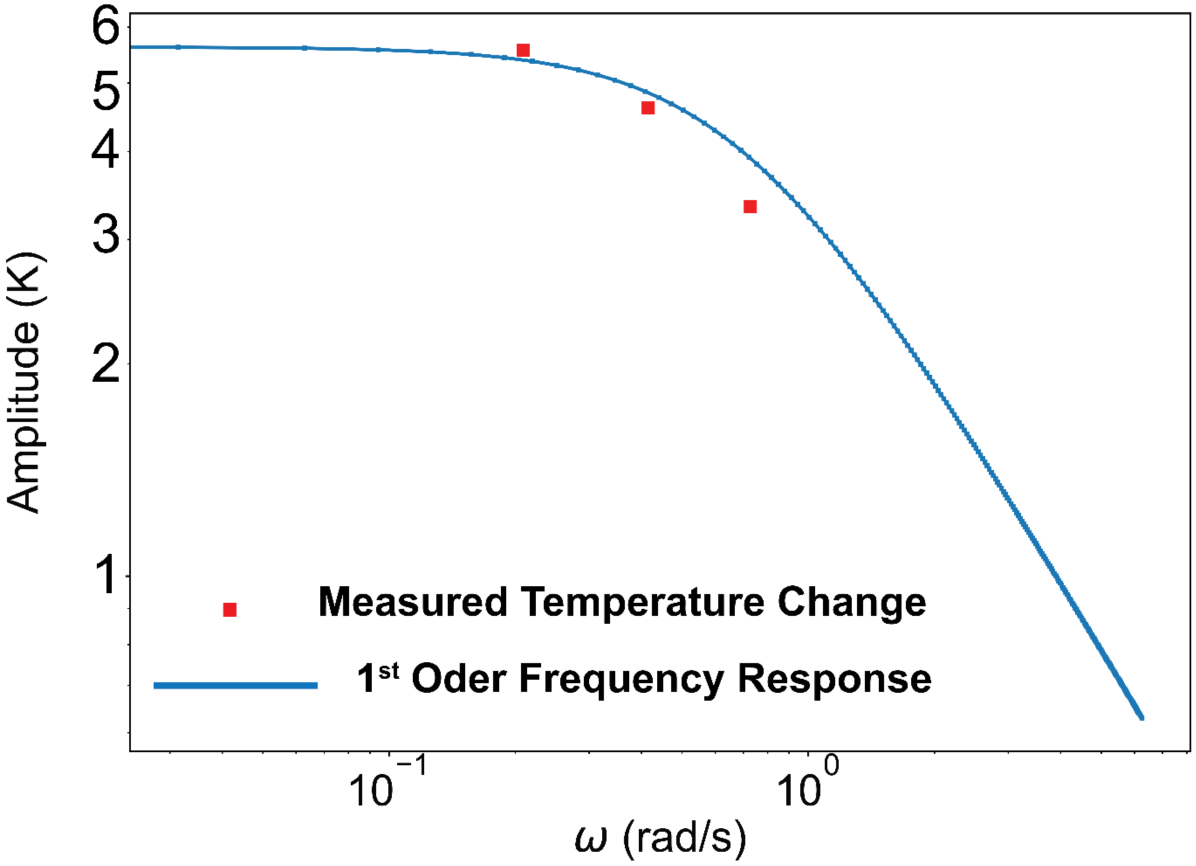

Figure S18. Frequency response of temperature dynamics of diamond sample. 1st order frequency response fitted with response time constant: $A/\sqrt{1+(\omega\tau)^2}$ where τ=0.71s is the time constant extracted from Figure S13, compared with the temperature change measured under different temperature tuning rates with rotation speeds of polarizer at ω=0.206 rad/s, 0.415rad/s and 0.728rad/s.

Reference

[1] H. M. Linares Rosales, P. Duguay-Drouin, and L. Archambault, Med. Phys. **2019,** 46, 2412-2421.
