# Supplementary figures and images for: Widefield Diamond Quantum Sensing with Neuromorphic Vision Sensors
Source: Adv Sci (Weinh). 2023 Nov 8;11(2):2304355. doi: 10.1002/advs.202304355 (PMC10787069; doi:10.1002/advs.202304355)

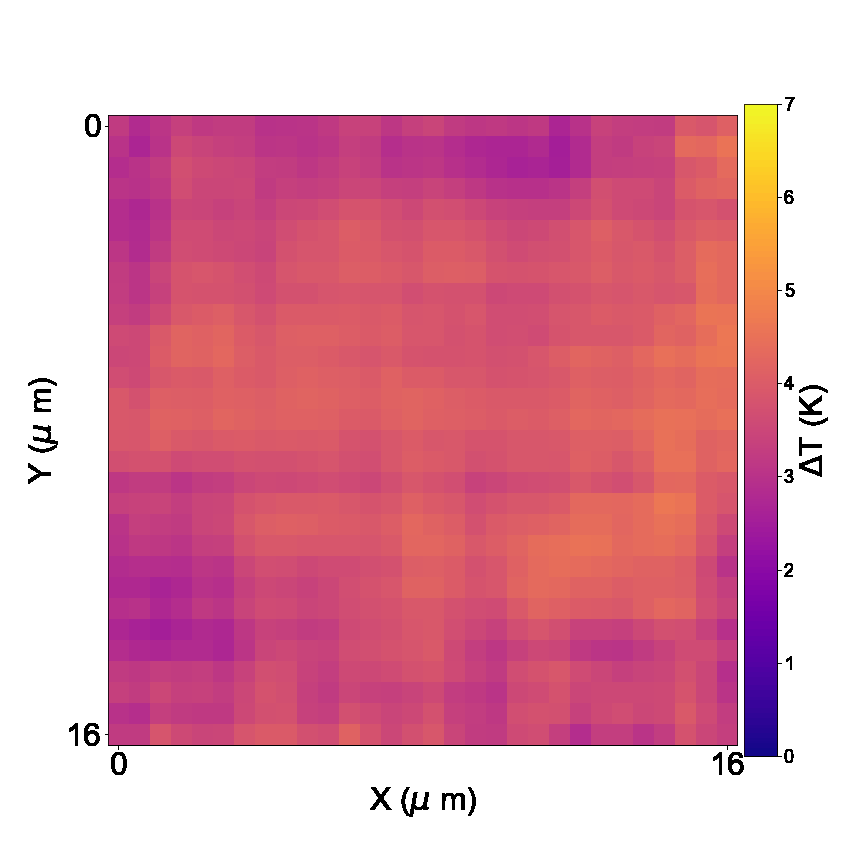

Supplement: Supplementary file 3 — Supplemental Movie 2 [file ADVS-11-2304355-s002.gif]
